# A refined, minimally invasive, reproducible ovine ischaemia–reperfusion–infarction model using implantable defibrillators: Methodology and validation

**DOI:** 10.1113/EP091760

**Published:** 2024-12-19

**Authors:** Charlene Pius, Barbara Niort, Emma J. Radcliffe, Andrew W. Trafford

**Affiliations:** ^1^ Division of Cardiovascular Science, School of Medical Science, Faculty of Biology Medicine and Health, University of Manchester Manchester Academic Health Science Centre Manchester UK

**Keywords:** echocardiography, electrocardiography, heart disease, infarction, ischaemia, reperfusion, troponin

## Abstract

Ischaemic heart disease remains a leading cause of premature mortality and morbidity. Understanding the associated pathophysiological mechanisms of cardiac dysfunction arising from ischaemic heart disease and the identification of sites for new therapeutic interventions requires a preclinical model that reproduces the key clinical characteristics of myocardial ischaemia, reperfusion and infarction. Here, we describe and validate a refined and minimally invasive translationally relevant approach to induce ischaemia, reperfusion and infarction in the sheep. The novelty and refinement in the procedure stems from utilization of implantable cardiac defibrillators prior to coronary engagement, balloon angioplasty to induce infarction, and intra‐operative anti‐arrhythmic drug protocols to reduce adverse arrhythmic events. The protocol is readily adoptable by researchers with access to standard fluoroscopic instrumentation, and it requires minimally invasive surgery. These refinements lead to a substantial reduction of intra‐operative mortality to 6.7% from previously published values ranging between 13% and 43%. The model produces key characteristics associated with the fourth universal definition of myocardial infarction, including ECG changes, elevated cardiac biomarkers and cardiac wall motility defects. In conclusion, the model closely replicates the clinical paradigm of myocardial ischaemia, reperfusion and infarction in a translationally relevant large animal setting, and the applied refinements reduce the incidence of intra‐operative mortality typically associated with preclinical myocardial infarction models.

## Abstract (highlights)

- **What is the central question of this study?**
  We sought to develop and describe in detail a minimally invasive, reproducible large animal model of myocardial ischaemia–reperfusion–infarction with improved survival outcomes and high translational relevance.
- **What is the main finding and its importance?**
  Adopting minimally invasive balloon angioplasty approaches, we developed a reproducible mode of ischaemia–reperfusion–infarction in sheep that satisfies key criteria of the fourth universal definition for myocardial infarction. Using an implantable cardiac defibrillator and intrasurgical anti‐arrhythmic drug routine, intra‐operative mortality was reduced by ≥50% in comparison to other large animal models of myocardial infarction.

## 1 INTRODUCTION

Cardiovascular diseases, mainly ischaemic heart diseases, are the leading cause of mortality in the UK (Cheema et al., 2022) and worldwide (WHO, 2023). Coronary artery disease is one of the most important causes of ischaemic heart disease (Sawyer & Vasan, 2017) and is characterized by a reduction in the volume of perfusion to the heart (i.e., ischaemia) or even its complete cessation (i.e., infarction) (Ambrose & Singh, 2015; Fuster, 1994; Saleh & Ambrose, 2018). Acute myocardial infarction (MI) is defined as myocardial necrosis in the context of myocardial ischaemia, which can be transmural, involving all three layers of the heart (endocardium, mid‐myocardium and epicardium), or non‐transmural, typically sparing the epicardium (Ambrose & Singh, 2015; Collet et al., 2021; Fuster, 1994; Greulich et al., 2019; Reimer et al., 1977; Saleh & Ambrose, 2018; Sarafoff et al., 2013). Classically, non‐transmural MI presents clinically with non‐ST‐segment elevation on the ECG and is treated primarily with anti‐platelet treatment, peri‐interventional anticoagulant treatment and coronary angiography, with a view to revascularization within 72 h (Collet et al., 2021). Transmural MI is associated with ST‐segment elevation (STEMI) on the ECG and is treated with primary percutaneous coronary intervention (PPCI). Complications following MI include ventricular arrhythmias (Bhar‐Amato et al., 2017; Goldman & Schafer, 2011) and heart failure (Torabi et al., 2008), which can manifest early or late (Cahill & Kharbanda, 2017; Takada et al., 2019; Torabi et al., 2008). Ventricular arrhythmias can occur in the form of ventricular tachycardia (VT) (Bhar‐Amato et al., 2017; Henkel et al., 2006) or ventricular fibrillation (VF). These life‐threatening complications necessitate increased research to identify new therapeutic targets that might ultimately alter prognosis following MI, and ultimately this necessitates translationally relevant animal models.

When studying a disease, especially the precise molecular aspects of dysregulation, the animal model should ideally be as clinically relevant as possible. Occluding a coronary artery in an experimental animal can induce a reaction comparable to an acute MI caused by atherosclerosis or thromboembolic events. Most of the current small (mouse or rat) (Sjaastad et al., 2002; Stafford et al., 2021) and large (pig, sheep or dog) mammal models of cardiac dysfunction from ischaemia consist of the permanent ligation or occlusion of the left anterior descending (LAD) coronary artery (Dardenne et al., 2013; Dib et al., 2006; Hegyi et al., 2018; Kim et al., 2002; Locatelli et al., 2011; Markovitz et al., 1989; Rabbani et al., 2008; Spata et al., 2013; for review see Riehle & Bauersachs, 2019; Sattler et al., 2019). Although they are reliable models for inducing tissue damage and heart failure, they do not accurately reflect the clinical setting occurring as a result of a reperfusion of the occluded vessel during PPCI. Other models use intracoronary injections of thrombogenic material, causing a permanent occlusion, and therefore, in the same way, do not reflect the typical clinical situation. Importantly, the reperfusion phase can also be associated with cardiac dysfunction known as ischaemia–reperfusion injury, including arrhythmias, myocardial stunning and vascular obstruction (Ibanez et al., 2017). None of these potential sequelae occurs in models of total and permanent occlusion.

The development of percutaneous transluminal coronary angioplasty balloon catheters to treat coronary atherosclerotic stenosis has led to the creation of less invasive ischaemia–reperfusion animal models. However, mortality in these models ranges from 13% to 43% (Bowen et al., 2001; Ikram et al., 1997; Spata et al., 2013), predominantly owing to arrhythmic death or a low cardiac output state (Ikram et al., 1997). This high mortality rate is at odds with the key principles of animal research of reduction, refinement and replacement, referred to within the UK as the 3Rs and promoted by the National Centre for the Replacement, Refinement and Reduction of Animals in Research (https://nc3rs.org.uk) (Lee et al., 2020; Percie du Sert et al., 2020), making the establishment of a large mammal MI model more challenging.

The heart of large animals shows many electrophysiological and contractile similarities to humans (Milani‐Nejad & Janssen, 2014), which is why modelling cardiac diseases in these species generally better reflects human pathologies, and thus drug and interventional effects, than in small animals (Houser et al., 2012). Additionally, large animal models better allow for the implementation of minimally invasive approaches and use of clinical‐grade materials and devices, thus conferring an inherent refinement aspect of 3Rs considerations. Canine, porcine and ovine models using balloon occlusion of coronary arteries already exist (e.g., Amado et al., 2004; Charles et al., 2000; Ishikawa et al., 2014). However, both porcine and canine models present limitations. Studies suggest that the vascular architecture of the porcine and canine heart differs from that of a human heart, and thus might be less representative of the remodelling that occurs in a human with ischaemic heart disease. Specifically, when compared with human, pig and sheep hearts, rodent and canine hearts are known to have a greater collateral network, which can affect the ability to form a predictable infarct size (Flameng et al., 1979; Piktel & Wilson, 2019; Shin et al., 2021). Furthermore, pig models are associated with a high rate of sudden death caused by ventricular arrhythmias following MI, as reviewed by Munz et al. (2011). Lastly, an additional logistical consideration is that husbandry can be challenging with certain breeds of pig that grow rapidly to large size. With the exception of the miniaturized Gottingen minipig (∼18 kg), commonly used pig breeds include the Yucatan minipig (>80 kg) and Yorkshire or Large White (> 300 kg), and these are substantially heavier at maturity than the Welsh sheep used in the present study. The ovine heart is one of the closest to the human heart, and therefore is accepted as a good preclinical animal model for cardiovascular research (Milani‐Nejad & Janssen, 2014). A clear scientific protocol for the experimental induction of ischaemia–infarction–reperfusion injury and study of STEMI in sheep is lacking. It is essential to develop comprehensive, reproducible, reliable protocols and criteria for knowledge and skill transfer, and to ensure that investigations can be replicated with minimal animal suffering to ensure good proximity to the clinical scenarios that the in vivo modelling is attempting to reproduce.

Here, we have developed a reproducible, minimally invasive large mammal ischaemia–infarction–reperfusion model in sheep, with a considerably lower mortality rate (6.7%) than other studies and therefore presenting a ≥50% reduction in mortality in comparison to the best‐performing comparable studies in other previously reported large animal models. To that end, we: (1) refined a prophylactic intra‐operative anti‐arrhythmic drug protocol; and (2) incorporated a precoronary engagement surgical step involving the implantation of an internal cardiac defibrillator, with the purpose of being able to deliver rapid defibrillation of intra‐operative ventricular arrhythmias.

## 2 MATERIALS AND METHODS

### 2.1 Ethical approval

All procedures involving the use of animals were performed in accordance with the United Kingdom (UK) Animals (Scientific Procedures) Act, 1986 and European Union Directive 2010/63 (PP5868419). Local ethical approval was obtained from the University of Manchester Animal Welfare and Ethical Review Board. Reporting of animal experiments was in accordance with the ARRIVE guidelines 2.0 (Percie du Sert et al., 2020).

### 2.2 Animals

Experiments were performed on naive adult (∼18‐month‐old) female Welsh Mountain sheep (30 animals, 38 ± 6.5 kg). Animals were not randomized, because this study focused on model development and did not contain a sham‐operated arm. Given these consideratons, all statistical comparisons were paired to presurgical values in the same animals. Animals were group housed, fed hay and water ad libitum, and maintained on a 12 h–12 h light–dark cycle for ≥1 week prior to surgical intervention.

### 2.3 Myocardial infarction surgery

In line with all experiments necessitating the use of general anaesthetic, animals were fasted overnight to prevent the risks associated with gastric distension but had unrestricted access to water. The full step‐by‐step protocol is available in the Extended Methods in the Supporting information. Induction of anaesthesia was achieved using a combination of isoflurane (5% vol/vol; Baxter Healthcare, UK), nitrous oxide (50% vol/vol) and O_2_ (50% vol/vol at a combined flow rate of 10 L/min) administered via facemask. The depth of anaesthesia was confirmed by loss of the corneal reflex (Siddiqui & Kim, 2022). To facilitate passage of an endotracheal tube into the trachea, lignocaine local anaesthesia spray (Xylocaine, Astra Zeneca, UK) was applied topically, followed by tracheal intubation with a curved endotracheal tube (8–10 mm). The cuffed endotracheal tube was then inflated to ensure a sealed circuit, avoid anaesthetic leakage and prevent secretions and gastric contents from entering the lungs. The tube was then connected to a mechanical tidal ventilator (Zoovent, UK) and ventilation performed at a rate of 15 breaths/min. The maintenance of anaesthesia was achieved using an isoflurane concentration of ∼3% mixed with O_2_ (4 L/min). The following parameters were monitored constantly during the surgery: (1) the depth of anaesthesia, by assessing the corneal blink reflex and lack of response to stimuli; (2) arterial blood pressure, using an electronic sphygmomanometer (Cardell Veterinary Monitor 9402, Sharn, USA) placed on the shaved tail base; (3) arterial O_2_ saturation (kept at >95%), using a Doppler pulse oximeter (Nonin Medical, USA) placed on the tongue or on the shaved ear; and (4) the ECG, using a five‐lead continuous ECG (EMKA Technologies) digitized to a computer at a sampling rate of 1 kHz (IOX2, EMKA Technologies).

In order to correct for any blood loss during the surgery, an i.v. maintenance fluid (0.9% NaCl, Baxter, USA) was administrated, via an 18‐ to 22‐gauge cannula (BD Microlance, UK) positioned in the lateral saphenous vein of the right hindlimb, at a constant flow rate over the course of surgery. This venous line was also used as a route for the i.v. administration of drugs (Figure S1), via a coupled three‐way tap.

Meloxicam (0.5 mg/kg s.c.; Metacam, Boehringer Ingelheim, Germany) and amoxicillin (20 µg/kg i.m.; Betamox, Norbrook, UK; or amoxicillin sodium i.m., Bowmed Ibisqus, UK) were used to provide analgesia and antibiosis. The surgery was carried out in two stages following aseptic conditions.

### 2.4 Implantation of the internal cardiac defibrillator

The first stage in the refined protocol consists of the implantation of an internal cardioverter defibrillator (ICD; Medtronic; DF1 or DF4 configuration) device in order to allow prompt cardioversion of any sustained intra‐operative ventricular arrhythmias and record the electrical cardiac function in the postoperative period. In detail, the animal was positioned in left lateral recumbency. A sterile field was prepared by shaving the wool over the right cervical region and cleansing with iodine (iodinated povidone 7.5%; Videne, UK). An ∼8 cm skin incision was made in the jugular groove, and blunt dissection was used to expose the jugular vein. Once adequately freed of attached fascia, 2–0 silk sutures (Ethicon, USA) were loosely placed proximally and distally. Further blunt dissection was then carried out to identify the carotid artery, which runs deeper, alongside the vagus nerve. The carotid artery was then freed from the vagus nerve using blunt dissection, and 2–0 silk anchoring sutures were placed loosely at the proximal and distal points of the artery (Figure S1).

After tying off the proximal part of the jugular vein, a small incision was made into the vein and maintained open using a vein pick to allow the insertion of an ICD lead (Medtronic, USA) into the right ventricular apex under fluoroscopic guidance (BV Pulsera, Philips, The Netherlands). The correct lead tip position was assessed prior to fixation in the right ventricle by connecting the lead to an electrophysiological analyser (Medtronic 2090 Analyser) and assessing three intra‐operative ECG parameters: (1) the pacing threshold (i.e., the lowest pulse amplitude at which the heart can still be paced); (2) intracardiac potential (R‐wave amplitude); and (3) lead impedance (300–1500 Ω) (Rajappan, 2009; Swerdlow et al., 2020). Satisfactory lead positioning was confirmed by a combination of: (1) pacing threshold <1 V; (2) R‐wave amplitude >6 mV; and (3) lead impedance <1500 Ω. Once satisfactory lead and pacing parameters were confirmed, the ICD lead was connected to the ICD body and secured in position using 2–0 silk suture where it entered the jugular vein, which minimizes the risk of dislodgment. Any excess lead length was wrapped in loose loops around the ICD before being positioned inside a subcutaneous pocket created above the jugular distal to the original incision directed toward the right shoulder. The pocket was closed securely using Vicryl 2–0 sutures. To avoid inappropriate shocks, the device was configured to detect only the following zones: VT, VF and Fast VT zones). In this ovine model, inappropriate T‐wave detection has been observed as high sinus tachycardia rates and T‐wave over‐sensing; therefore, the device was programmed purely for detection. The defibrillator was then set to emergency mode for the duration of the surgery, allowing for fast manual defibrillation when intra‐operative ventricular arrhythmias occurred.

### 2.5 Induction of myocardial infarction

Access to the arterial circulation was obtained via the carotid artery, which was identified at the start of the surgery. The 2–0 suture originally placed at the proximal end of the vessel was secured. Using the Seldinger technique (Seldinger, 1953), the carotid was punctured with a 14‐gauge cannula. A soft, curved‐tip guidewire (Abbott, USA) was then inserted through the cannula and advanced into the arterial lumen. The guidewire was held securely in place whilst the cannula was withdrawn. A 12 cm, 6 Fr haemostatic introducer sheath (Abbott, USA) was passed over the guidewire into the lumen. The guidewire was withdrawn, leaving the introducer sheath in the vessel. The sheath was loosely secured with a suture placed into the surrounding soft tissue to avoid displacement owing to carotid pulsation. About 10,000 IU of heparin sodium (Wockhardt, India) was injected as a bolus, and the i.v. maintenance fluid in the right posterior limb cannula was switched to one containing 0.9% NaCl i.v. infusion with 10 IU heparin/mL. The medication protocol, including the prophylactic anti‐arrhythmic drug regime, is summarized in Figure S2.

A 6 Fr Judkins right (JR4) catheter (Runway, Boston Scientific, Quincy, MA, USA) was preloaded with a 0.014 inch / 0.35 mm J‐tipped wire (Cordis, USA or Abbott, USA) and advanced through the introducer sheath to the aortic valve level under fluoroscopic guidance, leading with the J‐tipped wire. We used a Judkins right catheter because, in our experience, this gave better anatomical engagement with the left ostia than left variants.

The wire was then removed, and the left main stem was engaged with a 50:50 mix of radiocontrast agent (Visipaque, GE Healthcare, USA; 270 mg/mL) and 0.9% NaCl solution injected through the sheath catheter. A coronary angiogram was performed to opacify the left coronary system. The left coronary system consisted of the left main stem, which bifurcates into the left circumflex coronary artery and the LAD coronary artery, also known as the left homonymous in sheep (Markovitz et al., 1989; Rabbani et al., 2008). The left circumflex coronary artery supplies the posterior and lateral free walls of the left ventricle (LV) whereas the LAD coronary artery supplies the anterolateral, septal and apical walls of the LV (Locatelli et al., 2011; Markovitz et al., 1989). Upon identification of the LAD coronary artery, a hydrophilic 0.014 inch guidewire (Abbott, USA) was advanced to the distal LAD coronary artery. Depending on the calibre of the vessel, an appropriately sized intracoronary balloon catheter (Apex Monorail or Emerge, Boston Scientific) was advanced and positioned within the LAD coronary artery, after the second diagonal branch (Figure S3). The length of the intracoronary balloon used was 20–40 mm, with a width ranging from 2.00 to 2.75 mm depending on the coronary anatomy and calibre determined empirically during coronary angiography using the Judkins catheter diameter as a guide. The balloon catheter was inflated to the specified recommendations using an Indeflator (BasixCompak inflation device, Merit Medical, USA), with a repeat coronary angiogram thereafter confirming complete occlusion of flow distal to the inflated balloon (Figure S3). The balloon remained inflated for 90 min, occluding blood flow distal to the balloon, hence creating the infarct.

ECG and intracardiac electrogram (via ICD) parameters were monitored continuously for the presence of ST elevation, T‐wave changes, conduction abnormalities and ventricular arrhythmias. ST‐segment changes were usually observed within minutes of coronary occlusion, with evidence of additional ventricular activity seen ∼30–40 min into the occlusion. These ranged from the occasional ventricular ectopic (VE) beats to VF requiring defibrillation. In the event of a persistent ventricular arrhythmias, such as VT or VF, the animal would be defibrillated promptly via the ICD to terminate the arrhythmia.

About 90 min after inflation, the balloon was deflated, and coronary blood flow distal to the occlusion site was confirmed with a repeat coronary angiogram (Figure S3). The intracardiac equipment and sheath were removed. The distal carotid artery was tied off with a 2–0 silk suture. The wound was closed in three layers, closing the muscular fascia, the subdermal tissue and, finally, the cutaneous layer. During the wound closure, the isoflurane concentration was gradually reduced from 3% to 0%. The endotracheal tube was left in place until the animal showed the signs of a rejection reflex (i.e., swallow or cough). The recovery from anaesthesia was monitored closely for evidence of respiratory distress and arrhythmias. The i.v. catheter was left in place until the animal was fully awake to allow immediate i.v. access. Once the animal was alert and all vital signs were within normal range, it was placed in a single‐housed postoperative recovery pen in full sight and communication with its peers and was given access to food and water.

The animal was monitored closely during the recovery period. It was considered fully recovered from anaesthesia when visibly alert, standing and having urinated. After 24 h of postoperative recovery housing (in sight and vocalization range of group‐housed peers), the animal was returned to group housing. No further intervention was carried out for ≥3 days to ensure complete recovery from surgery and to allow the ICD lead to settle in its intracardiac position. Animals were monitored regularly by trained personnel (at least once daily) to ensure adequate postoperative recovery.

### 2.6 In vivo assessments

To assess the evolution of various clinical and cellular parameters over time following MI, the animals were divided randomly into three groups (3‐day MI, 1–1.5 week MI and 8 week MI) to elucidate the temporal development of remodelling and wound healing associated with the early inflammatory (acute), intermediate reparative (latent) and proliferative and late maturation (chronic) postinfarction stages (Prabhu & Frangogiannis, 2016). In vivo measurements were performed at baseline, postoperatively, and at the end point (Figure S4). There were two types of evaluations performed: full assessments and expedited assessments. The full in vivo assessment included measurement of weight and blood pressure, recording of ECGs, imaging using transthoracic echocardiography, blood sampling, and external interrogation of an intracardiac device once implanted. The expedited assessment involved only blood sampling and interrogation of the device.

### 2.7 Blood sampling

Blood was collected pre‐operatively, at multiple time points intra‐operatively (carotid artery access, pre‐occlusion, 30 min post‐reperfusion and 90 min post‐reperfusion), and at 2–5 days, 1–1.5 weeks and 8 weeks postinfarction. Venous blood sampling was performed preferably from the right jugular vein, with the animal gently restrained, using a sterile aseptic non‐touch technique. Alternative sampling sites included the left jugular vein or cephalic veins. The skin was shaved and aseptically cleansed before sampling. Amongst the blood parameters evaluated were whole‐blood cardiac troponin I (cTnI; VetScan i‐STAT, Abaxis, UK) as a marker of myocardial necrosis, along with a full biochemistry profile (Skyla VB1 Analyser, Woodley, UK). Cardiac troponin I was determined using a point‐of‐care testing system (upper limit of detection, 50 ng/mL), which provided a result within 10 min.

### 2.8 Transthoracic echocardiography

Animals were positioned on their rump and maintained in a sitting position and, under gentle restraint, transthoracic echocardiography (Vivid, GE Healthcare, USA) was performed, with the probe positioned on the right side of the chest, with the right forelimb lifted to provide access to the thorax (Briston et al., 2011; Dibb et al., 2009). Image quality was optimized by shaving the chest and using an ultrasound transmission gel (Aquasonic, Germany) for better contact. Parasternal short‐axis (SA) and long‐axis (LA) views were used for evaluation of cardiac structure and function. The SA‐mid view was taken below the mitral valve level, where the papillary muscles were visible. The SA‐distal view was the most distally obtainable SA image of the LV to visualize the apex and adjacent region. In humans, regional wall motion abnormalities can be assessed using a 17‐segment ECHO model, in which the LV walls are divided into 17 segments that can be correlated with the blood supply (Lang et al., 2015).

For the analysis, images were exported and converted from DICOM to JPEG format using Microdicom Viewer (Microdicom, Bulgaria). The JPEG files were opened in Fiji ImageJ (National Institutes of Health, USA), and multiple measurements were taken.

In the short‐axis mid and short‐axis distal images, the anterior wall thickness measurement represented the infarcted region, and the posterior wall thickness represented the non‐infarcted region (Figures S5 and S6). Prior to the surgery, measurements were also taken from these identical sites to serve as baseline values. The fraction of wall thickness change was calculated using Equation (1) to determine the difference in wall thickness between the infarcted region and the non‐infarcted region. In order to assess the fractional area change as a measure of contractile function, the LV cavity area was also measured at mid and distal LV levels in systole and diastole and calculated using Equation (2).

(1) $$\mathrm{Fraction}\;\mathrm{of}\;\mathrm{wall}\;\mathrm{thickness}\;\mathrm{change}=\frac{\left(\mathrm{Noninfarcted}-\mathrm{Infarcted}\right)}{\mathrm{Non}\;\mathrm{infarcted}\;}$$

(2) $$\mathrm{Fractional}\;\mathrm{area}\;\mathrm{change}\;=\frac{\left(\mathrm{Diastolic}\;\mathrm{area}-\mathrm{Systolic}\;\mathrm{area}\right)}{\mathrm{Diastolic}\;\mathrm{area}\;}\;\times \;100$$

From the parasternal long axis images, the interventricular septal thickness, LV end‐diastolic diameter (LVEDD), LV end‐systolic diameter (LVESD) and LV posterior wall thickness were measured in diastole and systole, as shown in Figure S7. An adaptation of the simplified Quinones equation (Quinones et al., 1981) was used to calculate the ejection fraction (EF) from the LVESD, LVEDD and an apical contractility estimate seen from the SA‐distal views using Equation (3). In this equation, the *K* value (Table 1) represents the apical contractility. Additionally, fractional shortening (FS) was also calculated as another measure of contractile function using Equation (4) and expressed as a percentage.

(3) $$\mathrm{EF}=\frac{\mathrm{LVED}D^{2}\;-\;\mathrm{LVES}D^{2}\;}{\mathrm{LVED}D^{2}}\;\times \;100+K$$

(4) $$\mathrm{FS}=\frac{\mathrm{LVEDD}-\mathrm{LVESD}}{\mathrm{LVEDD}}\;\times \;100$$

**TABLE 1:** *K* values (apical contractility measurements expressed as percentages), used in Equation (3).

Apical contraction	*K* value (%)
Normal	+10
Hypokinetic	+5
Akinetic	0
Dyskinetic	−5
Apical aneurysm	−10

### 2.9 Electrocardiography

Surface ECGs were recorded at intervals. Perioperative ECG recordings (EMKA Technologies, France) were performed in the seated position using five surface electrodes in an orthogonal arrangement on shaved and cleaned sites to allow better contact. The identical electrode position was used again intra‐operatively, but the animal was in left lateral recumbency during surgery. Upon connecting the electrodes, the animal was allowed some time to settle, thus minimizing any potential stress‐related ECG changes or motion artefacts. A 10 min ECG was then recorded through IOX software (EMKA Technologies, France)

For the analysis, the files were opened in ECG Auto (EMKA Technologies, France) and converted to a text file (*.TXT) format to allow the files to be opened on LabChart (AD Instruments, UK). The recordings were smoothed and filtered to reduce noise and breathing artefacts using a 21‐point (Bartlett) window and a 2 Hz high‐pass and 50 Hz notch filter. The software generated multiple averaged traces consisting of 10 consecutive beats from a 1 min selected recording period where intervals could be selected manually.

### 2.10 Euthanasia

The sheep were initially heparinized (10,000 IU i.v.), then euthanased with an anaesthetic overdose (pentobarbitone sodium, 200 mg/kg i.v.), with death confirmed through permanent cessation of the circulation. Animals were euthanased at one of three predetermined time points post‐MI: 3 days, 1.5 weeks and 8 weeks post‐MI.

### 2.11 Statistical analysis

All data are expressed as the mean ± SD. Statistical analysis was performed using Prism v.10 (GraphPad Software, San Diego, CA, USA). Data were first tested for normality using Shapiro–Wilk, Kolmogorov–Smirnov, Anderson–Darling and D'Agostino–Pearson omnibus in GraphPad Prism. Where data were not normally distributed, data were transformed using a natural logarithm, log_10_, reciprocal, square root or exponential, depending on skew (Ennos, 2007). Normally distributed data were analysed using Student's *t*‐test, one‐way ANOVA, repeated‐measures one‐way ANOVA and mixed‐effects model analysis (Caldwell et al., 2014). Where data were not normally distributed despite transformation, an equivalent non‐parametric test was used. The relationship between two variables was determined using simple linear regression, and the correlation was evaluated using Pearson's correlation. Data were considered significant if the *P*‐value was <0.05, and *P*‐values to *P* < 0.0001 are given in full.

## 3 RESULTS

### 3.1 Developing a large mammal model of infarction–reperfusion

Following left coronary artery angiography, the angioplasty balloon was successfully placed below the second diagonal branch of the LAD coronary artery (Figure S3). Only 2 of the 30 sheep died after MI induction from intractable VF unresponsive to ICD cardioversion. Total mortality was therefore 6.7%, with all deaths occurring intra‐operatively under anaesthesia before reperfusion.

Using a minimally invasive coronary angiographic technique, the infarct was created by inflating an intra‐coronary balloon for 90 min to occlude blood flow, followed by reperfusion. Both occlusion and reperfusion were confirmed by angiography. In order to validate the model, we sought to achieve the key criteria set out in the fourth universal definition, where MI is defined as myocardial necrosis in the context of myocardial ischaemia resulting in an elevation of a cardiac biomarker, such as cTnI (Daubert & Jeremias, 2010), with at least one value above the 99th percentile upper reference limit. The diagnosis of MI requires additional criteria, including at least one of the following: (1) clinical symptoms of ischaemia, including chest discomfort; (2) ECG ischaemic features, such as the development of new changes in the ST and T segment (ST‐T) or new left bundle branch block; (3) new pathological Q‐waves; (4) imaging evidence of reduced cardiac wall contractility; and/or (5) evidence of an intracoronary thrombus visualized via angiography or on autopsy (Thygesen et al., 2018, 2012). Specific objective assessment of chest discomfort is clearly impossible to achieve in animal models; however, each of the remaining four criteria are assessable with readily applicable methods or investigations. We will, in turn, consider each of the assessable fourth universal definition criteria achieved using the minimally invasive model of STEMI presented here.

### 3.2 Temporal changes in serum cTnI

**FIGURE 1:**
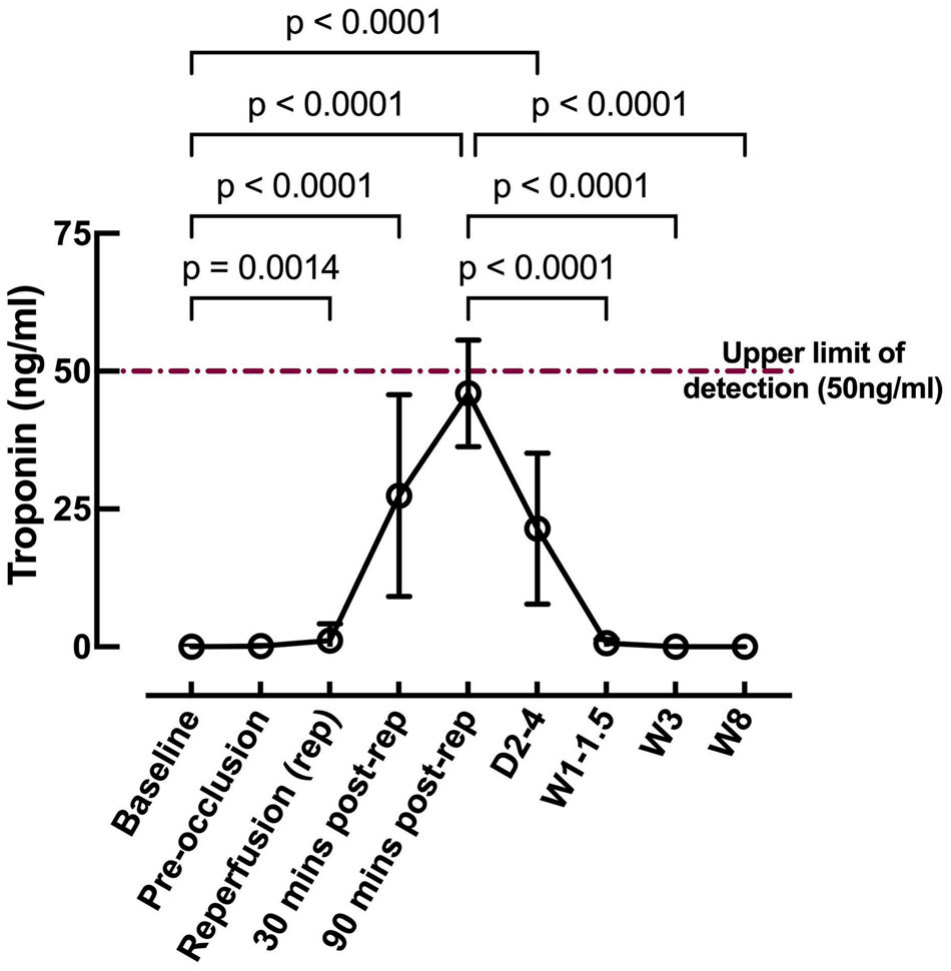
Cardiac troponin I measurements at baseline, intra‐operatively and on days 2–4 and at 1–1.5, 3 and 8 weeks, demonstrating the expected rise‐and‐fall pattern seen in myocardial infarction. Number of animals (*n*): baseline = 28; pre‐occlusion = 28; immediate reperfusion = 27; 30 min post‐reperfusion = 28; 90 min post‐reperfusion = 28; days 2–4 = 28; weeks 1–1.5 = 20; week 3 = 9; week 8 = 10. Kruskal–Wallis test.

Measurements were made at various stages during the procedure (Figure 1). All animals had near‐zero values at baseline after ICD lead placement but before coronary engagement (0.03 ± 0.1 ng/mL). After inflation of the angioplasty balloon, we observed an elevation in cTnI (*P* < 0.0001), displaying the typical rise‐and‐fall pattern seen in STEMI patients (Dasgupta & Wahed, 2013; Mahajan & Jarolim, 2011; Maynard et al., 2000; Morrow et al., 2007). The cTnI levels increased significantly during reperfusion (1.15 ± 3 ng/mL; *P* = 0.0014) and continued to rise at 30 min post‐reperfusion (27.4 ± 18.3 ng/mL; *P* < 0.0001), with a peak observed at a measurement time of 90 min post‐reperfusion (46 ± 9.7 ng/mL; *P* < 0.0001) when compared with baseline (0.03 ± 0.1 ng/mL). The levels started to decline by days 2–4 (21.5 ± 13.7 ng/mL; *P* < 0.0001) with a further decline after 1 week (0.64 ± 0.7 ng/mL; *P* < 0.0001 vs. peak) and returning to baseline values by week 3 (0.03 ± 0.03 ng/mL) and remaining low until the end of the study (week 8; 0.007 ± 0.02 ng/mL) (Figure 1).

### 3.3 Electrocardiographic changes following myocardial ischaemia–reperfusion injury

**FIGURE 2:**
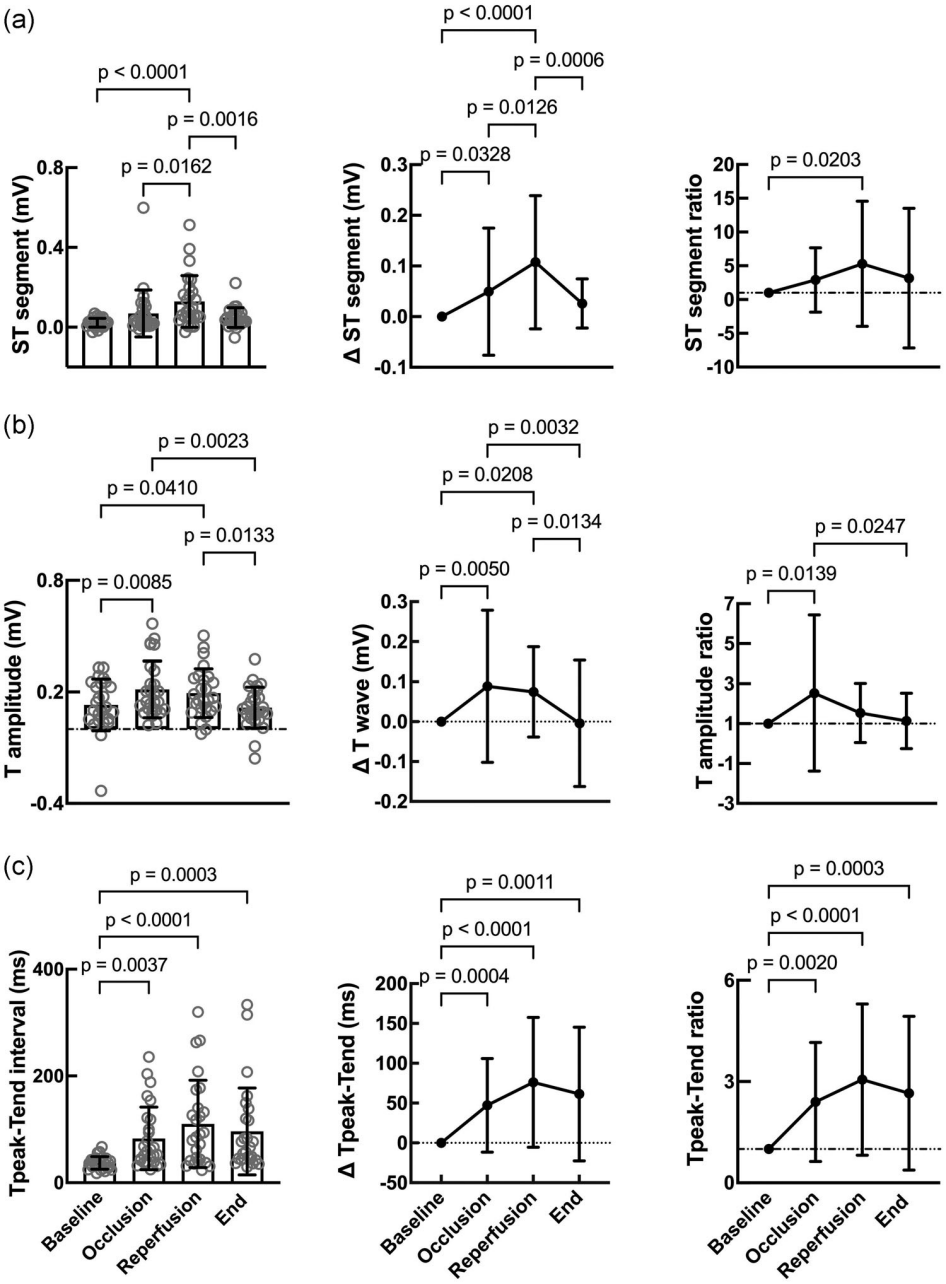
Electrocardiographic changes following ischaemia–reperfusion injury. Summary data showing (from left to right): absolute measurement; change in parameter compared with baseline; and ratio of change in parameter normalized to baseline, for: (a) ST segment; (b) T‐wave amplitude; and (c) T_peak_–T_end_ interval between peak and return to baseline of the T wave. Number of animals (*n*): baseline = 25–27; occlusion = 25–27; reperfusion = 25–26; end = 25–26. Repeated‐measures one‐way ANOVA.

The intention with this study was to create a preclinical ischaemia–reperfusion–infarction model with ST‐segment elevation MI, thus most closely resembling the clinical cohort of patients being treated with PPCI. The ST segments started to rise upon coronary occlusion (0.07 ± 0.1 mV), with a statistically significant increase seen on reperfusion (0.13 ± 0.13 mV; *P* < 0.0001) compared with baseline (0.02 ± 0.02 mV). ST‐segment normalization was already evident at the end of surgery (0.05 ± 0.05 mV; *P* = 0.0016 vs. reperfusion; Figure 2a). The dynamic pattern of these ST segments supports the diagnosis of MI (Marti‐Carvajal et al., 2015; Thygesen et al., 2018). Intra‐operative ventricular arrhythmias (VF) requiring intracardiac defibrillation were observed in 8 of 27 animals and typically developed ∼30 min after coronary occlusion. Notably, there were no changes in blood potassium levels (baseline, 5.47 ± 0.42 mmol/L; 90 min postocclusion, 4.99 ± 1.15 mmol/L; 8 weeks postocclusion, 5.24 ± 0.47 mmol/L).

The T‐wave amplitude peaked on reperfusion (0.19 ± 0.13 mV; *P* = 0.04) compared with baseline (0.13 ± 0.14 mV), with a gradual return to near normal values by the end of surgery (0.12 ± 0.02 mV; *P* = 0.013 vs. reperfusion; Figure 2b). The T‐wave peak amplitude increment could be a consequence of interstitial hyperkalaemia from myocardial ischaemia (Weiss et al., 2017).

We also took the opportunity to calculate the T_peak_–T_end_ interval, which is suggested as a measure of transmural dispersion of repolarisation (Xia et al., 2005). Upon coronary occlusion, the T_peak_–T_end_ interval more than doubled (83 ± 59 vs. 37 ± 12 ms; *P* = 0.0037), with maximal prolongation seen on reperfusion (110 ± 82 ms; *P* < 0.0001), declining by the end of surgery yet remaining prolonged compared with baseline (96 ± 81 ms; *P* = 0.0003; Figure 2c).

### 3.4 Change in cardiac contractile function and planimetry

**FIGURE 3:**
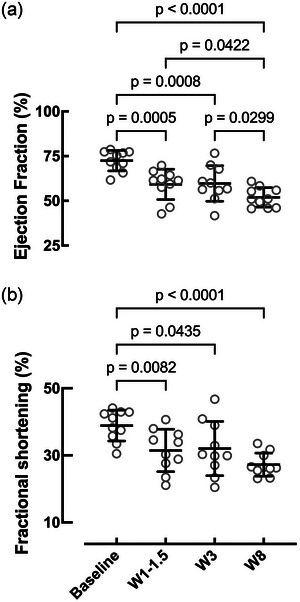
Echocardiographic evaluation of left ventricular systolic function: Measurements of ejection fraction (a) and fractional shortening (b) from baseline to week 8. Number of animals (*n*): baseline = 25; W1–1.5 = 17; W3 = 10; W8 = 10. Mixed‐effects analysis.

Left ventricular EF was estimated at intervals, as described in the Materials and methods section. Presurgery EF was 73% ± 6% and declined following MI, being 58% ± 2.1% at 1–1.5 weeks (*P* < 0.0001), 60% ± 3.1% at 3 weeks (*P* < 0.05) and 52% ± 5.5% at 8 weeks post‐MI (*P* < 0.0001), demonstrating fractionally a 29% ± 10% decline in EF compared with baseline (*P* < 0.0001; Figure 3a). Although the final EF value at 8 weeks was higher than those mentioned in other permanent ligation models (Li et al., 2013), the absolute reduction in EF of 21% was larger than the 14% described by Locatelli et al. (2011). We also measured FS, as a measure of contractility, and this parameter also declined from presurgical levels of 39% ± 5% to 31% ± 6% at 1–1.5 weeks and 27% ± 3% at 8 weeks respectively following MI (Figure 3b; *P* < 0.0001).

After an MI, it is known that LV remodelling involves changes in wall thickness in both infarcted and non‐infarcted regions (Hassell et al., 2017; Locatelli et al., 2011; Sutton & Sharpe, 2000). We first evaluated the wall thickness of the infarcted region in comparison to the non‐infarcted LV wall. Here, we present the results as the fraction of the change in wall thickness at the infarcted site by comparing wall thickness with the non‐infarcted site on the same acquisition plane in systole and diastole. Here, positive values indicate wall thinning in the infarcted region relative to the non‐infarcted region. This approach was adopted to reduce the effect of any inconsistencies in the acquisition plane, particularly at the distal LV level, where landmarks were less clear.

**FIGURE 4:**
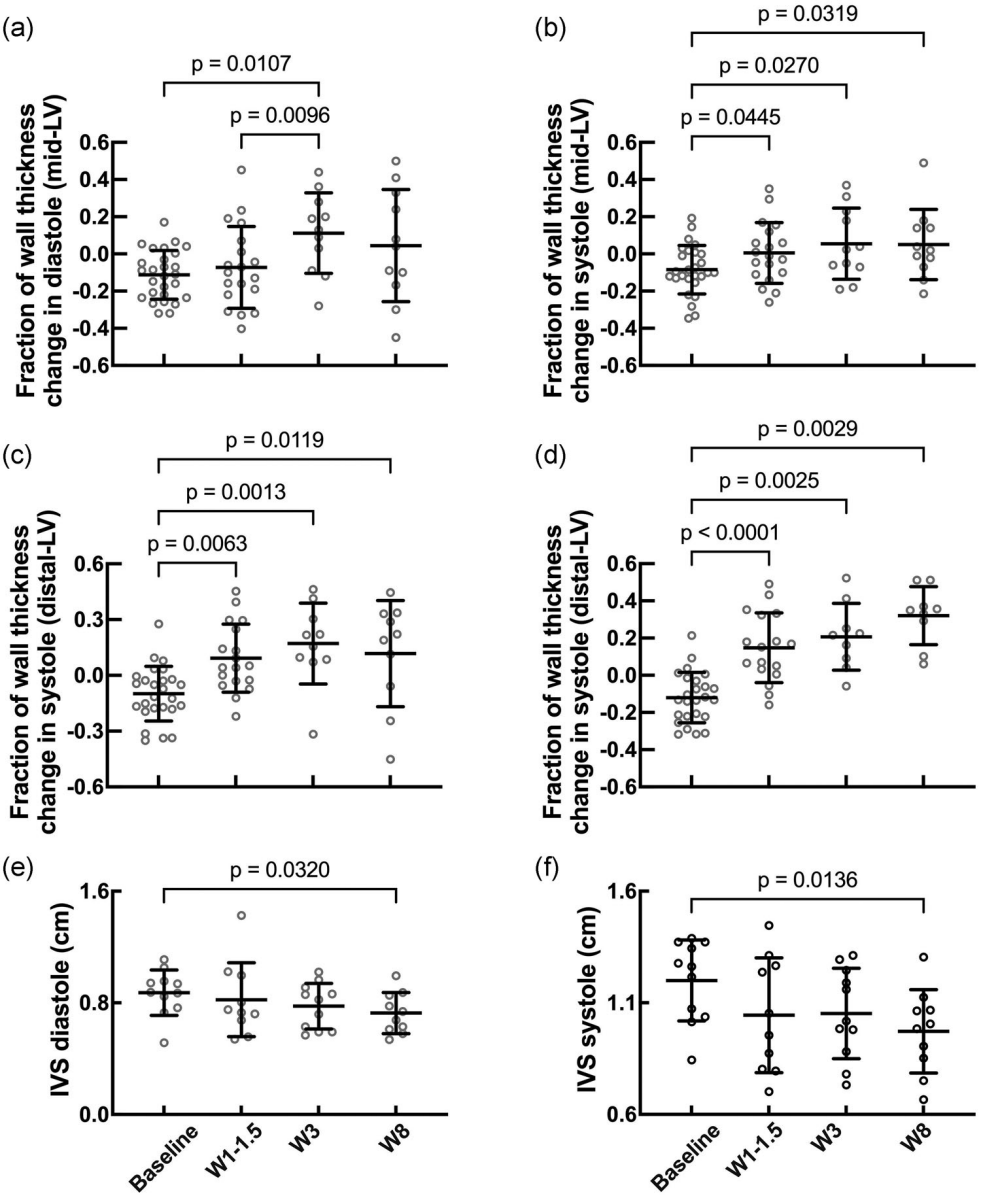
Temporal evolution of changes in wall thickness postinfarction. (a,b) Changes in wall thickness at mid‐level of the left ventricle (LV) in diastole (a) and systole (b). Number of animals (*n*): baseline = 27; W1–1.5 = 19; W3 = 11; W8 = 11. (c,d) Changes in wall thickness at distal LV level in diastole (c) and systole (d). (e,f) Absolute measurements of wall septal wall thickness are shown at diastole (e) and systole (f). Number of animals (*n*): baseline = 27; W1–1.5 = 19; W3 = 11; W8 = 11. Mixed‐effects analysis.

At mid‐LV level, there were no statistically significant changes in the fractional wall thickness change in diastole (Figure 4a) or systole (Figure 4b) following MI, which was probably attributable to the more apical infarct location. Conversely, at the distal‐LV level, where the infarct would be more pronounced, an increase in fractional wall thickness change was noted at weeks 1−1.5 and week 3 in both diastole and systole compared with baseline (Figure 4c,d). In systole, there was an increase in fractional wall thickness change, which was maintained from 1–1.5 to 8 weeks post‐MI (Figure 4d). These changes are probably attributable to the occurrence of eccentric hypertrophy in the non‐infarcted wall, thinning of the infarcted wall segments or a combination of both. These differences were more pronounced in systole because the infarcted segment is akinetic and unable to thicken adequately compared with the hypercontractile non‐infarcted segment (Rechavia et al., 1995). Additionally, a thinning of the interventricular septal absolute wall thickness was noted 8 weeks post‐MI in both diastole and systole (Figure 4e,f). These wall thickness changes further support the finding of regional wall motion abnormality, which further fulfils criteria set out in the universal definition of MI.

### 3.5 Gross morphology and infarct size

**FIGURE 5:**
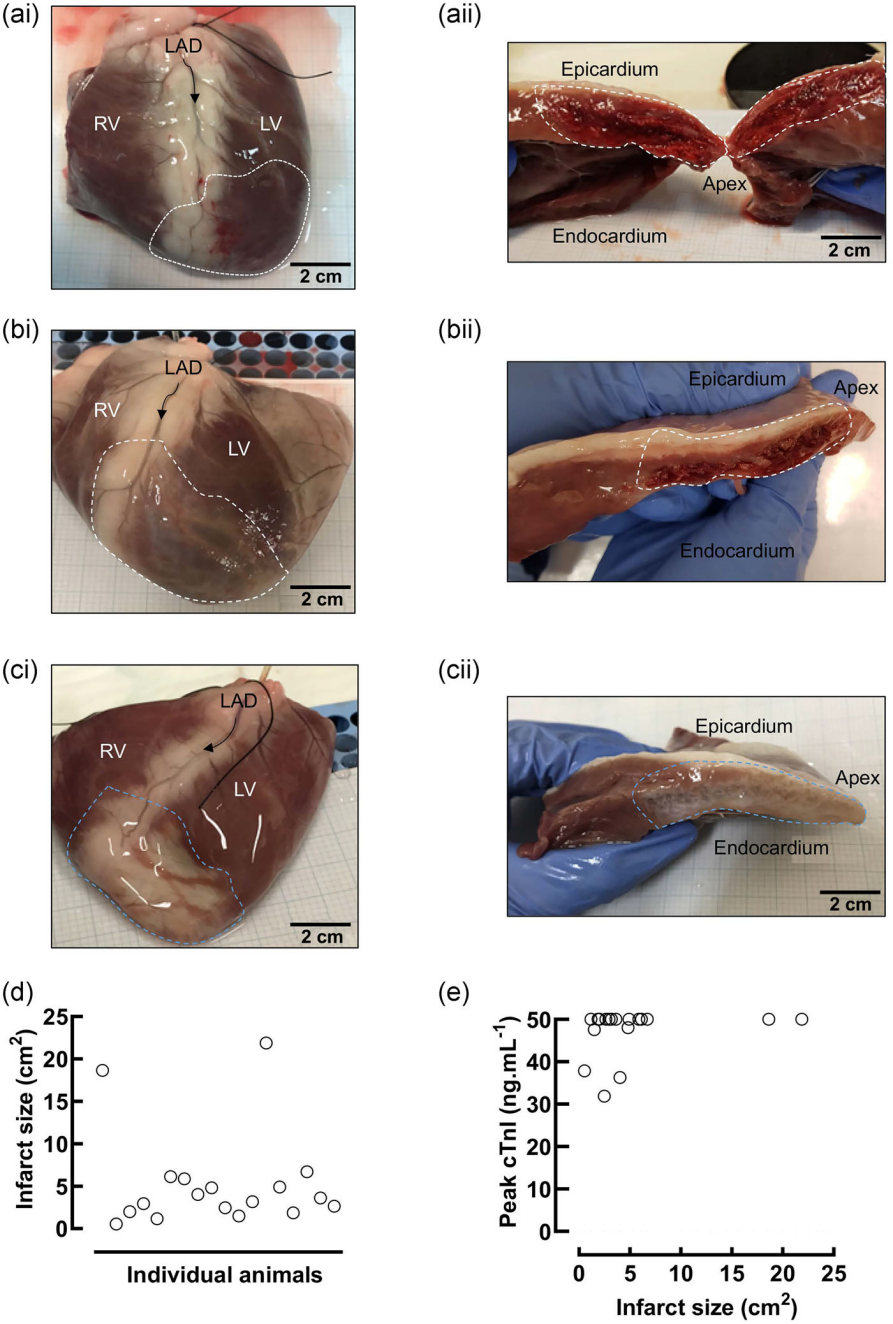
(a–c) Images of infarcts at 3 days (a), 1.5 weeks (b) and 8 weeks (c) as whole‐ventricle images (i) and left‐ventricular cross‐sectional images (ii), demonstrating different appearances in the infarct over time. The scale bar is shown in black. (d) Infarct sizes from all animals at 3 days, 1.5 weeks and 8 weeks. (e) The peak troponin value plotted against the infarct size, with no relationship seen (*n *= 26). Abbreviations: cTnI, cardiac troponin I; LAD, left anterior descending; LV, left ventricle; RV, right ventricle.

The transient occlusion of the LAD after the second diagonal branch resulted in a well‐defined area of infarction detectable at all three time intervals, despite varying appearance reflecting the acute, proliferative and maturation stages of scar formation (Figure 5a–c). At 8 weeks, infarcts were more easily measured owing to the presence of a visible white area of scar tissue. The infarcts were less well defined visually after 3 days and 1.5 weeks. The infarct was best seen in a cross‐sectional image, revealing the intra‐ and transmural nature of scar tissue distribution. Planimetry was used to assess the size of the infarct (Figure 5d) from the anterior surface of the LV. The average size of an infarct was 4.7 ± 1 cm^2^. However, the infarct frequently extended around the apex into the posterior wall, making it difficult to determine the infarct size accurately using this measurement approach.

The variability in infarct size is consistent with the variable infarct size seen in humans. No correlation between infarct size and troponin levels (Figure 5e) was observed, but this is probably related to the limitations on both infarct size measurement and the upper limits of detection of the point‐of‐care cTnI assay (50 ng/mL).

## 4 DISCUSSION

We aimed to create a refined, minimally invasive ischaemia–infarction–reperfusion model of myocardial infarction with improved survival outcomes. It was crucial that our model survived to the specified time points of 3 days, 1.5 weeks and 8 weeks in order to assess the temporal evolution of the acute (inflammatory), latent (reparative) and chronic (maturation) phases following a myocardial infarction. In order to achieve this, the experimental protocol was refined with specific measures to reduce the mortality rate previously described in the literature (Ikram et al., 1997), including the inclusion of prophylactic anti‐arrhythmic medications (Latini et al., 1984; Li et al., 2013; Marti‐Carvajal et al., 2015) and the implantation of an internal cardiac defibrillator. Using this surgical and medication protocol, we successfully created reproducible infarcts and reduced procedural mortality. Importantly, the model fulfils the key criteria of the fourth universal definition of MI, demonstrating: (1) the rise and fall of a cardiac biomarker (cTni); (2) ECG changes in the ST‐T segment; (3) ECG evidence of wall motion abnormality; and (4) evidence of scar/infarct on the heart.

During the occlusion period, we observed cardiac repolarisation abnormalities in the form of ST‐T segment changes. The presence of ST elevation indicates a transmural infarction, which affects the heterogeneity of the ionic properties of cardiac cells from the epicardial, myocardial and endocardial layers (Deshpande & Birnbaum, 2014; Okada et al., 2020). It is debateable whether the repolarisation abnormalities are more reflective of transmural injury or the baso‐apical position of the injury (Di Diego & Antzelevitch, 2014).

We also observed changes in contractile function and wall thickness over the temporal evolution of the MI. It is known that, during the post‐infarct remodelling phase, the infarcted segment undergoes thinning and expansion. The apico‐anterior segments are particularly vulnerable to this because they are the thinnest segments, with the greatest curvature (Galli & Lombardi, 2016; Litwin et al., 1994; Pennock et al., 1997; Pfeffer & Braunwald, 1990). This is accompanied by hypertrophy of the non‐infarcted region (Galli & Lombardi, 2016; Litwin et al., 1994; Pennock et al., 1997; Pfeffer & Braunwald, 1990; Rubin et al., 1983; Sutton & Sharpe, 2000), which might serve as a temporary compensatory mechanism. The hypertrophy occurs in response to increased wall stress as a consequence of the infarcted segment (Sutton & Sharpe, 2000). This is often not enough to restore the original function, as we have demonstrated here. In fact, this eccentric hypertrophy often contributes to worsening dilatation during remodelling (Galli & Lombardi, 2016), which might explain the deterioration in LV function observed at 8 weeks.

There was large inter‐animal variability in infarct size, with a coefficient of variation of 104%. This is probably attributable to inter‐animal anatomical variations in the LAD coronary artery at the D2 bifurcation point and the limitations of the infarct measurement method. In an effort to standardize the approach, we aimed to occlude the LAD coronary artery after the D2 bifurcation. However, the variability in coronary anatomy, particularly of the LAD vessel, which has been previously described in sheep, is likely to have contributed to this (Locatelli et al., 2011). We aimed to achieve an anterior‐apical infarct involving ∼25% of the LV. In some animals, the point of D2 bifurcation was relatively proximal and would affect more than the intended area of myocardium, whereas in other animals, the D2 branch followed a similar course to the LAD coronary artery towards the apex, potentially co‐supplying the intended infarct territory.

With the described refinements in our experimental protocol, our approach was associated with a lower rate of attrition and improved mortality rate in comparison to existing work (Bowen et al., 2001; Ikram et al., 1997; Spata et al., 2013). Thus, we were able to follow the temporal evolution of MI successfully at an in vivo level spanning the acute, latent and chronic phases, which will allow future cellular and tissue studies.

Finally, we demonstrated that this model is clinically relevant owing to similarities with existing STEMI patients undergoing PPCI reperfusion treatment. As a result, this model will allow for a better understanding of the pathological evolution following MI and will help in the research of new therapeutic targets that might improve patient outcomes post‐MI. Furthermore, with further refinement, this model might be able to reflect a translatable heart failure model of ischaemic cardiomyopathy following STEMI and could be used to select cardioprotective medications to protect STEMI patients from reperfusion damage.

The refinement aspects developed in this study encompassed the inclusion of a prophylactic intra‐operative anti‐arrhythmic strategy, including the use of amiodarone, lidocaine and atenolol and the use of an implantable cardiac defibrillator. Although approximately one‐third of animals developed ventricular arrhythmias requiring defibrillation, our overall intra‐operative mortality was reduced to 6.7%. This compares favourably with previous studies (Bowen et al., 2001; Munz et al., 2011; Sattler et al., 2019; Spata et al., 2013) and provides a methodological approach that is easily applied to other large animal ischaemia reperfusion studies. The presented approach affords an improvement in procedural survivability over other ovine infarction models, including micro‐embolization (13%) (Ikram et al., 1997), intracoronary alcohol injection (29%) (Rienzo et al., 2020), intracoronary thrombogenic coil placement (30%) (Charles et al., 2000) and a model of balloon reperfusion without ICD placement (22%) (Charles et al., 2000). However, open‐chest permanent ligation models in sheep appear to have higher survivability (100%) (Locatelli et al., 2011; Rabbani et al., 2008). Importantly, however, these models are both more invasive, requiring a thoracotomy and thus more intensive postoperative management, and are considerably less representative of the infarction–reperfusion situation observed clinically following percutaneous interventions. Likewise, rapid pacing models of dilated cardiomyopathy in sheep have nearly 100% survival (Briston et al., 2011; Dibb et al., 2009; Horn et al., 2016; Lawless et al., 2019; Rademaker et al., 2012) but are representative of a much rarer clinical cause of heart failure than MI and fail to reproduce key features associated with infarction‐induced cardiac dysfunction, thus potentially limiting their potential translational relevance.

### 4.1 Study limitations

The present study does have some limitations, including the following. First, only female sheep were used because common farming practices mean that mature male sheep were unavailable. There are recognized sex‐related differences in animals and humans in both physiological and pathophysiological situations. It would be of interest to elucidate any sex‐dependent effects in future studies. Second, A five‐lead rather than 12‐lead ECG was used throughout, and therefore we are unable to comment in full on changes in electrical activity of the septal, anterior or lateral walls of the left ventricle. Additionally, our assessment of ST‐segment changes might therefore not fully reflect the extent of changes that occurred intra‐operatively. Third, owing to the post‐procedural use of the hearts, we were unable to perform Triphenyltetrazolium chloride (TTC)/Evans Blue assessments of infarct size. Equally, cardiac magnetic resonance with or without late gadolinium enhancement would have provided more definitive measurements of infarct size and/or the area at risk. Currently, our animal facility does not have access to such imaging equipment, and the use of such modalities would be of great future benefit for the longitudinal assessment of disease progression and subject stratification. Fourth, we did not determine the precise cause of VF during the occlusion phase of the procedure. Although we did not detect changes in circulating potassium levels, we cannot exclude the possibility that tissue hyperkalaemia owing to cell death is a major factor driving arrhythmia induction. Fifth, an absolute decrease of 21% in EF was observed 8 weeks postinfarction and was greater than that reported in other sheep infarction models; it could be argued this is a model of heart failure with preserved EF. However, in keeping with clinical observations, it is likely that cardiac function would continue to deteriorate with extended durations postinfarction.

Whilst the use of ICDs affords an opportunity to: (1) remotely detect abnormal ventricular rhythms requiring defibrillation during coronary occlusion; (2) maintain surgical site sterility during delivery of defibrillation shocks; (3) record ECGs remotely; and (4) deliver programmed electrical stimulation protocols, there are limitations to their use. Principal amongst these is the high cost associated with ICDs. In this study, we were able to use resterilized, out‐of‐date devices marked ‘not for human use’ and provided free of charge.

## AUTHOR CONTRIBUTIONS

All work was completed at The University of Manchester. Conception—Andrew W. Trafford conceived experiments and secured funding. Design—Andrew W. Trafford, Charlene Pius. Acquisition, analysis, interpretation—Charlene Pius led the model development and performed all analyses; all authors contributed to surgical and in vivo procedures. Drafting, revision and critical evaluation—Charlene Pius, Barbara Niort and Andrew W. Trafford drafted the manuscript. All authors reviewed, critiqued and approved the final version of the manuscript and agree to be accountable for all aspects of the work in ensuring that questions related to the accuracy or integrity of any part of the work are appropriately investigated and resolved. All persons designated as authors qualify for authorship, and all those who qualify for authorship are listed.

## CONFLICT OF INTEREST

The authors have no financial or competing interests to declare.

## Supporting information



Online Data and Methods Supplement

Supplementary videos 1–3

## Data Availability

Metadata associated with this study are available via a Creative Commons CC BY 4.0 licence from https://doi.org/10.48420/27078391.
