# Supplementary material for: A refined, minimally invasive, reproducible ovine ischaemia–reperfusion–infarction model using implantable defibrillators: Methodology and validation
Source: Exp Physiol. 2024 Dec 19;110(2):215–29. doi: 10.1113/EP091760 (PMC11782204; doi:10.1113/EP091760)
Supplement: Supplementary file 1 — Online Data and Methods Supplement [file EPH-110-215-s001.pdf]

Online Data and Methods Supplement

Supplemental Figures: Methodology

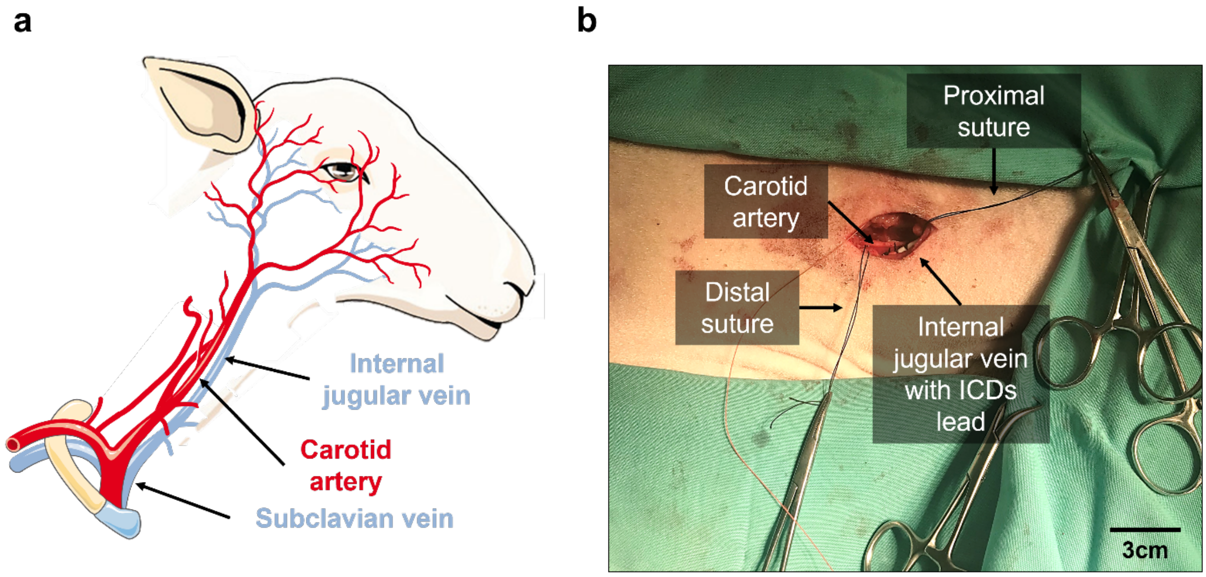

Supplemental Fig. 1 – Intraoperative images of surgery. a, Schematic of the isolated vessels in the sheep neck. **b,** Overview of the surgical site (right side of neck) shaved and cleaned, showing the incision with sutures placed on the distal and proximal aspect of the carotid artery.

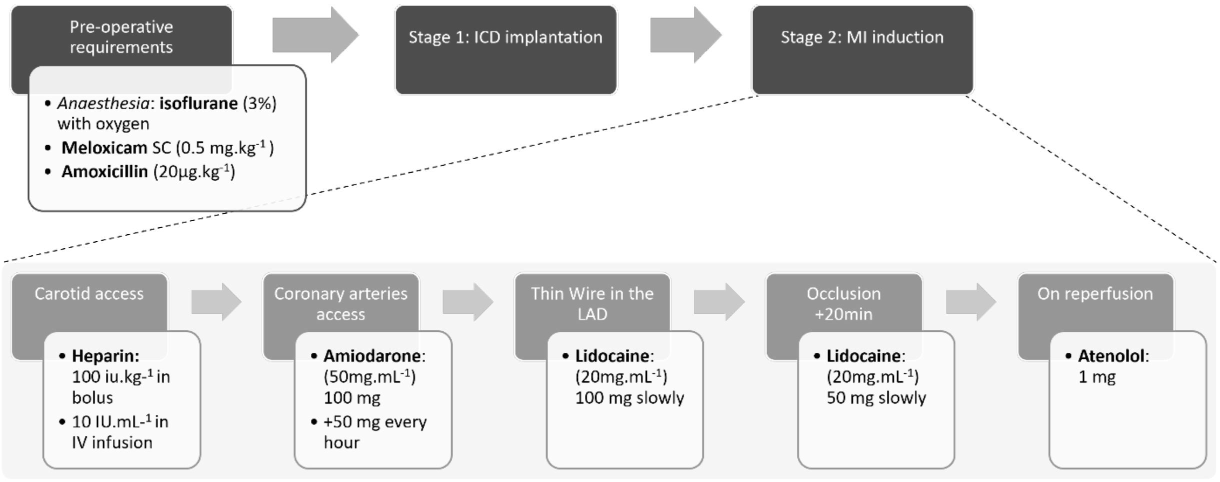

Supplemental Fig. 2 - Drug protocol for MI induction surgery. Diagram of the timings of medications administered during MI induction surgery. Internal cardiac defibrillator (ICD) device; anterior descending artery (LAD).

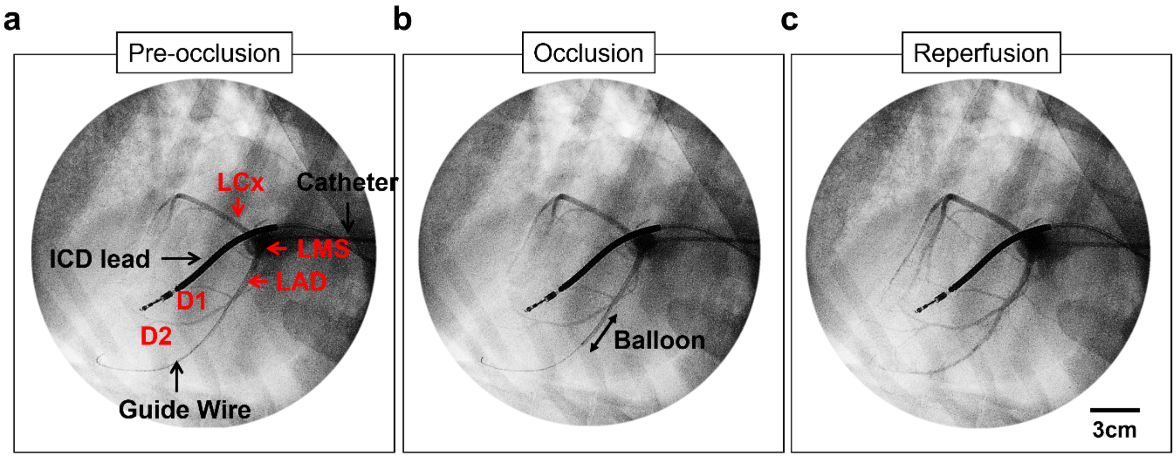

Fig. 3 - Fluoroscopic images during MI induction surgery. a, Angiogram of the left coronary system showing the left main stem (LMS) bifurcating into the left anterior descending (LAD) and left circumflex (LCx) coronary artery. The ICD lead is also seen within the RV. **b,** Repeat coronary angiography with an inflated intracoronary balloon occluding flow distal to the balloon. **c,** Confirmation of the correct reperfusion of the heart. D1 and D2, diagonal branches. RA, right atria. (also see Supplemental Videos 1-3)

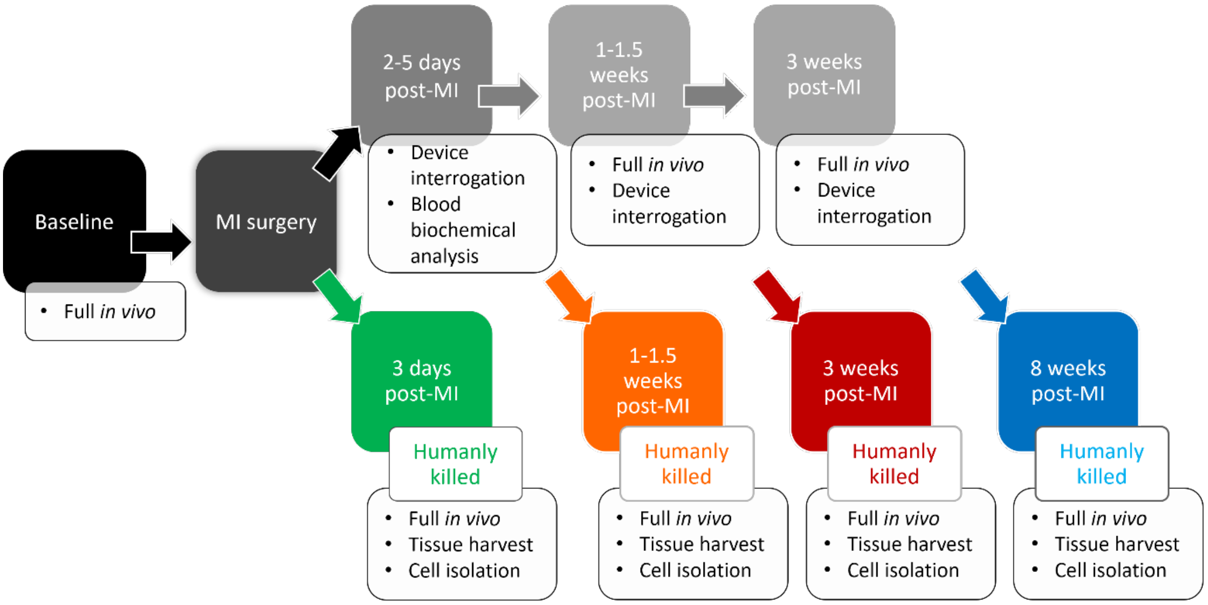

Supplemental Fig. 4 - *In vivo* schedule for 3 day, 1.5 week, 3 week and 8 weeks post MI. The full *in vivo* assessment included measurement of weight and blood pressure, recording of electrocardiograms, imaging using transthoracic echocardiography, blood sampling, and external interrogation of an intracardiac device once implanted.

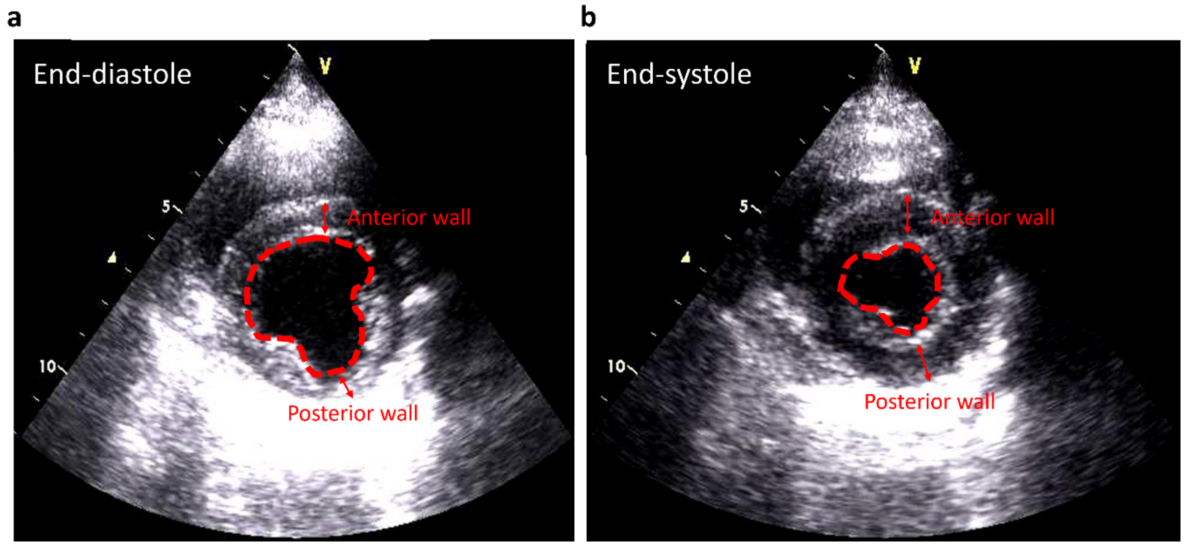

Supplemental Fig. 5 – Measurements from ECHO short axis mid views. Frames from the LV short axis views at mid-level in diastole **a,** and systole **b,** with the anterior and posterior wall thickness measurement sites (marked by red arrow) as well as the LV cavity area measurement (outlined in red-dotted line) shown.

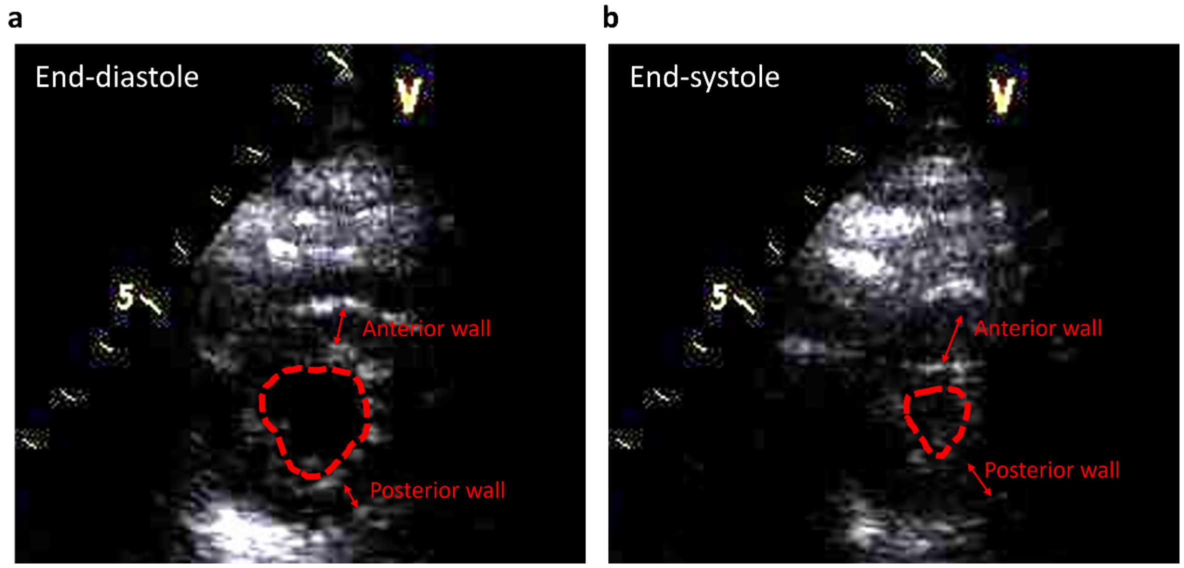

Supplemental Fig. 6 – Measurements from ECHO short axis distal views. Frames from the LV short axis views at distal-level in diastole **a,** and systole **b,** with the anterior and posterior wall thickness measurement sites (marked by red arrows) as well as the LV cavity area measurement (outlined in red-dotted line) shown.

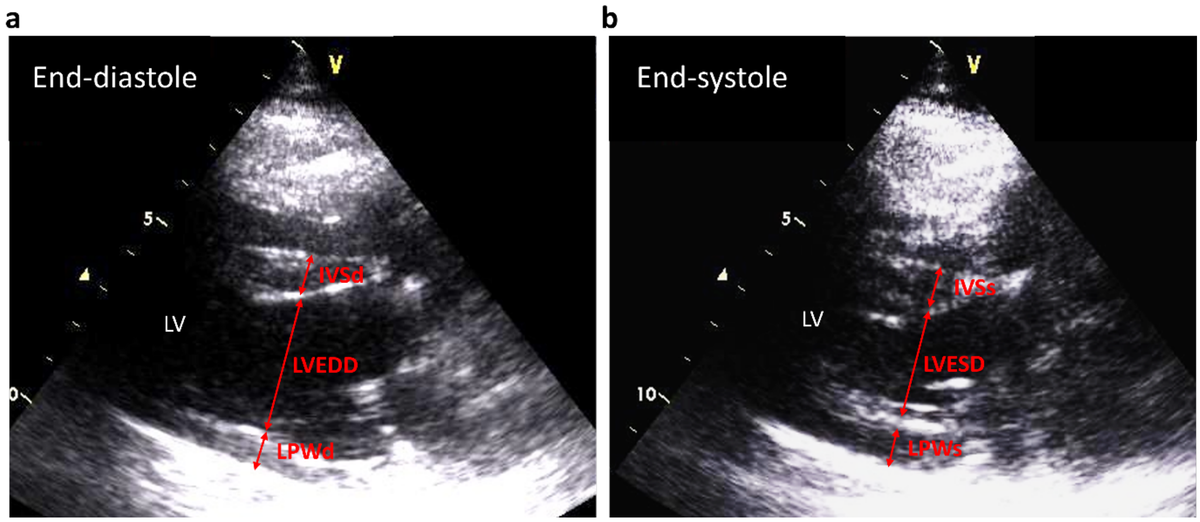

Supplemental Fig. 7 – Measurements from ECHO PLAX views. Frames from the PLAX views in diastole **a,** and systole **b,** with the interventricular septal thickness (IVS) (yellow arrows), LV end diastolic (LVEDD) and systolic diameter (LVESD) (blue arrows) and left posterior wall thickness (LPW) (orange arrows). For IVS and LPW measurements, the letter *s* or *d* at the end denote measurements taken in systole or diastole, respectively.

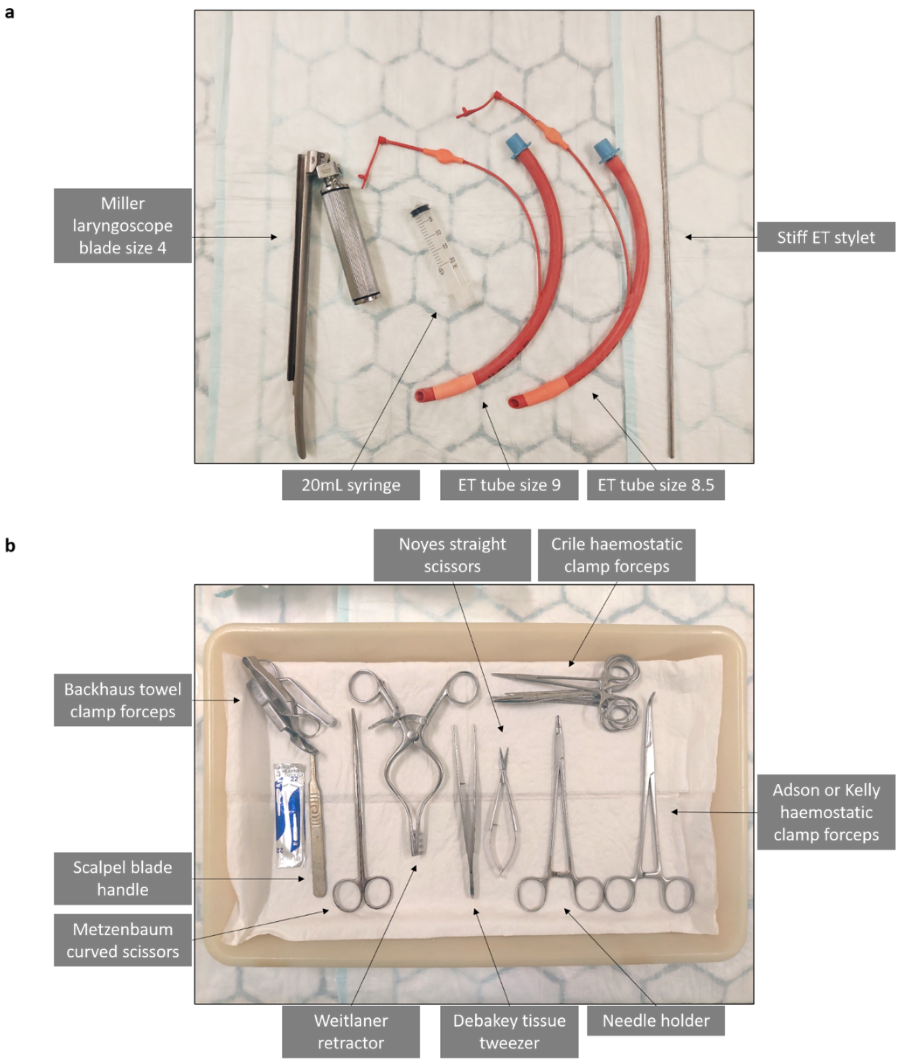

Supplemental Figure 8. – Typical surgical equipment.

a, Intubation equipment. **b,** Surgical tray.

Step by step guide to inducing myocardial infarction

MATERIALS

Animals

- Young, treatment naïve Welsh Mountain sheep (aged ~18 months) with an average weight 38.5 ± 6.5 kg
- Group housed, fed hay and water ad libitum and maintained in a 12 hour light/12 hour dark cycle for a minimum of 1 week prior to surgical intervention.

Reagents

- Lignocaine local anaesthesia throat spray (Xylocaine, Astra Zeneca, UK)
- x3 Heparin 10,000 IU in 10 ml vials (Wockhardt, UK)
- Contrast medium – Ionohexol (Omnipaque 300, GE Healthcare, USA)
- x3 Sodium chloride 0.9% 500ml and 1000ml (Baxter, USA)
- Prophylactic antibiotics (Amoxicillin 15 mg/kg) (Norbrook, UK)
- Prophylactic analgesia meloxicam (0.05mg/kg) (Norbrook, UK)
- Amiodarone (Hameln, UK)
- Lidocaine (Hameln, UK)

Premedication and Anaesthesia

- 1-chloro-2,2,2-trifluoroethyl difluoromethyl ether (isoflurane, Santa Cruz Biotechnology, USA)
- Oxygen and nitrous oxide (50:50) mix (BOC Healthcare, The Linde Group, Germany)
- Cone face mask rubber large

Equipment

- Hypodermic needle variable sizes 18, 20, 22G (BD Microlance, UK)
- 5ml, 10ml, 20ml and 50ml syringe (BD Plastipak, UK)
- IV infusion set (CareFusion, USA)
- 14G & 20G cannula (BD Venflon, USA)
- Blood pressure cuff and machine (Mindray, Australia)
- Pulse oximeter sensor (Mindray iMEC8 Vet Manuals, Mindray Bio-Medical Electronics Co., China)
- 5 lead ECG cable with crocodile clips and recording equipment, IOX software (EMKA Technologies, France)
- iSTAT Vetscan Hand held analyser and cTni cartridges (Abaxis, UK)
- Ultrasound transmission gel (Aquasonic, Germany)

- GE Vivid 7 echocardiography machine with 5S cardiac transducer (General Electrics, USA)
- Skyla VB1 Biochemistry analyser (Woodley, UK)
- C-arm fluoroscopy machine (BV Pulsera Mobile C-arm, Philips, UK)

Intubation (see Error! Reference source not found.

- Miller laryngoscope blade size 4
- Stiff endotracheal stylet
- Cuffed endotracheal tube (size 8.5 to 10; J.A.K Marketing, UK)
- 20 ml syringe (BD Plastipak, UK)
- The ribbon to tie the ET tube in place
- Bag valve mask

Monitoring and anaesthesia

- Anaesthetic machine (Zoovent, UK)
- Five-lead electrode cable with leg strips
- Electrocadiogram monitoring (IOX software, EMKA technologies,USA)
- Pulse oximeter sensor (Mindray iMEC8 Vet Manuals, Mindray Bio-Medical Electronics Co., China)
- Blood pressure cuff sized as tail cuff and BP recording equipment (Mindray, Australia)

Surgical site preparation, generic operative equipment & operator preparation

- Sheep clippers
- Iodinated povidone 7.5% (Videne, UK)
- Sterile drapes

Surgical tray (Error! Reference source not found.)

- Gallipot

- 2-0 Vicryl sutures (Ethicon,USA)
- 2-0 Silk sutures (Ethicon,USA)
- 2-0 Monocryl sutures (Ethicon,USA)
- Sterile gauzes
- Internal cardiac defibrillator
    - Generator (Medtronic, USA)
    - Right ventricular active fixation defibrillator leads (DF1 or DF4) (Boston Scientific, Medtronic, USA)
    - ICD compatible programmer with analyser cable and header (*e.g.* Medtronic 2090, Medtronic, Minnesota, USA)
    - PSA cables
- MI Induction
    - 14G cannula (BD Venflon,USA)
    - 12cm 6Fr haemostatic introducer sheath (Abbott Medical, UK), containing the sheath, a dilator and a mini-guidewire
    - 6F JR4 Guide catheter (Runway, Boston Scientific, USA)
    - Haemostasis valve (Honor,Merit Medical, USA)
    - Indeflator (balloon inflation device) with pressure monitor (BasixCompak inflation device, Merit Medical, USA)
    - 50ml syringe (BD Plastipak, USA)
    - Intracoronary balloon catheter, variable sizes 2.2 to 2.75mm diameter, 20-40mm length (Apex Monorail, Boston Scientific, MA, USA)
    - 0.35” J tipped guide wire (Cordis, USA)
    - 0.0014” intracoronary guide wire (Abbott, USA)

Operator preparation (Aseptic surgery)

- Surgical gown

- Surgical cap
- Sterile gloves
- Surgical face mask
- Radiation protective equipment – lead gown (Kenex, UK) and thyroid collar

Software

- ECG recording software details

Surgical procedure

A. Anaesthetic induction and preparation

⚠ Prior to surgery, animals are given access to water but food is witheld overnight to avoid intra-operative rumenal distension.

1. After pre oxygenation (100 % oxygen, ~ 3 – 5 mins), the animal is allowed to inhale a combination of oxygen and nitrous oxide in a 50:50 mix with isoflurane (3-5%) to induce anaesthesia.
2. Animal is lifted and placed on the preparation table in a sternal recumbency position, with the head elevated.
3. With the aid of a second operator, the jaw is opened with the head supported to allow the primary operator to spray two puffs of local anaesthetic (Lidocaine throat spray) into the back of the throat.
4. The facemask delivering oxygen nitrous 50:50 with isoflurane is replaced in preparation for intubation.
5. The facemask is removed and the jaw is held open with the head supported by the second operator. A laryngoscope is introduced and the vocal cords visualised.
6. A stiff stylet is taken down to the level of the vocal cords. An appropriately sized endotracheal tube is advanced over the stylet past the vocal cord and the stylet is removed. The cuff is inflated with air using a 20mL syringe and the tube is connected to a bag mask to confirm the appropriate tube placement in the trachea. The tube is then

connected to the ventilator. The appropriate tube placement is confirmed by appropriate chest wall movement with the ventilator, saturation recordings, and the maintenance of anesthesia, and the tube is secured with the tie around the jaw.

When advancing stylet, due care is taken not to advance the stylet too far to avoid damage to soft tissue or larynx. Adequate visualisation of vocal cords is required to ensure safe placement.

7. The animal was positioned in lateral recumbency for the rest of the procedure and positioned on the operating table. Administer pre operative analgesia and antibiotics of choice.
8. All monitoring equipment (saturations monitoring, ECG monitoring, and BP monitoring) is connected.
9. A 20G cannula is sited on the right hind leg for the purposes of administration of intraoperative medications. Pre-operative antibiotics and analgesia are administered at this stage.
10. The right side of the neck is shaved, providing a wide surgical field.
11. The skin is cleaned twice with an iodine-based antiseptic and draped.
12. Anatomical landmarks are palpated to delineate the jugular groove.
13. A 5 to 7cm incision is made to the skin with a blade within the jugular grove. The incision site is located two thirds of the way from the angle of the jaw to the shoulder tip. This is followed by blunt dissection down to identify the jugular vein.
14. The jugular vein is identified and freed. A proximal and distal 2-0 silk suture is placed loosely on the vessel. These ties are clipped onto the drapes using the Crile (?artery) forceps.

Gentle dissection is performed to free the jugular vein and carotid artery to avoid vascular damage. The vagal nerve must be gently freed from the carotid artery.

15. Further blunt dissection is performed deeper to identify the carotid artery, which runs alongside the vagus nerve. The carotid artery is freed from the vagus nerved and similar proximal and distal 2-0 silk sutures are placed and clipped to the drape loosely.

The procedure is carried out in two phases. The first stage involves implantation of an internal cardiac defibrillator to manage intraoperative life-threatening ventricular arrhythmias and the second stage is the induction of MI.

B. Implantation of an internal cardiac defibrillator (ICD)

1. The proximal jugular vein suture is tied off.
2. The mobile C-arm of the fluoroscopy machine is moved into position over the heart in the postero-anterior position.
3. With Noyes scissors, a venotomy is performed, exposing the inner lumen of the vessel.
4. With the aid of a vein pick, the vein is kept open and an active fixation RV lead, with a straight stylet in position, is advanced down to the right ventricular apex under fluoroscopy guidance. Due caution is taken when advancing the lead and lead advancement is stopped if there is any resistance.
5. As the lead crosses the tricuspid valve and is advanced into the RV, the ECG is monitored for the presence of ventricular ectopics suggestive of crossing the valve into the RV.

⚠ Advancement of the lead is performed cautiously across the tricuspid valve. If the lead does not directly cross the valve, the lead is prolapsed with the stylet retracted ~5cm - 8cm followed by straightening out the lead with the stylet fully inserted.

6. Once at the RV apex, the active fixation lead is deployed by applying clockwise turns on the proximal end of the lead to screw in the lead.
7. With the analyser connected to the distal end of the lead, the lead parameters are tested in the bipolar configuration. Target parameters include an R wave > 6mV, an impedance value between 300–1500Ω and a pacing threshold of <1V.
8. The lead is secured with 2-0 silk ties at the proximal and distal lead cuffs. The distal cuff is secured with the jugular vein simultaneously achieving vein closure and haemostasis.
9. The lead is connected to the appropriate port of the generator.
10. The subcutaneous pocket for the generator is created. The site needs to be sufficiently distal to the original incision towards the shoulder. The generator with the residual lead coiled is placed into the pocket.
11. The pocket is closed with interrupted 2-0 Vicryl sutures.
12. The VT, VF and FVT zones are programmed for detection only and all therapy is turned off. The rationale behind this is to avoid inappropriate shocks as the higher sinus rates and T wave oversensing in the model.

13. The device is then left connected wirelessly to the programmer in the emergency mode to allow prompt defibrillation of intra-operative life-threatening ventricular arrhythmias.

C. Induction of myocardial infarction

All the MI induction equipment (except the indeflator and the balloon) listed above should be pre-flushed with heparinised saline solution (i.e., 500mL sodium chloride 0.9% solution containing 10,000 IU heparin prepared in a sterile kidney dish) and prepared as follows:

- Insert the dilator into the 6 Fr haemostatic introducer sheath.
- Connect the 6F JR4 guide catheter to the Honor® Hemostasis Valve, which is connected to a 3-way valve, and then insert the 0.35” J tipped guide wire through the Hemostasis Valve all the way to the end of the catheter.

1. The previously identified carotid artery is the vascular access site for this part of the procedure.
2. The proximal suture is tied off.
3. The vessel is controlled with the previously placed proximal and distal ties.
4. A 14G cannula is advanced into the carotid artery towards the distal end.

The cannula is cautiously inserted to remain intraluminal and avoid transecting the carotid artery.

5. Once intravascular access is achieved, the x short guide wire is advanced down the cannula and exchanged for the 6F 12cm haemostatic sheath using the Seldinger technique.
6. The sheath is loosely secured via the side loop to prevent displacement secondary to the carotid pulsation.
7. Upon achieving arterial access, a 10,000 IU bolus if IV heparin is administered followed by a maintenance infusion of 10IU/ml to reduce the risk of thrombotic complications with the indwelling arterial equipment.
8. 30 minutes prior to coronary access, amiodarone 100mg IV is administered followed by a maintenance bolus dose of 50mg/hour.

9. Via the introducer sheath, a 6F Guide Judkins Right (JR4) catheter is advanced with a pre-loaded wire 0.35” 150cm J wire. This is introduced under fluoroscopic guidance with the J wire leading to reduce vascular trauma.

10. The J wire is advanced down to the aortic valve. When it reaches the aortic valve, mild resistance should be felt, which corresponds to the J wire looping at the valve level.

11. Then the guide catheter is advanced over the wire down to the aortic valve level and the wire is removed.

12. A 50ml syringe with 50% contrast mix is connected to the end of the guide catheter.

13. Using fluoroscopic guidance, the catheter is advanced into the left coronary system with catheter motion and contrast injection used to confirm/identify position.

    Engagement of left coronary artery system is done gently under direct fluoroscopic guidance observing catheter tip motion and a gentle contrast injection to ensure the ostium of the vessel is not dissected and to avoid deep intubation of the coronary.

14. Once engaged adequately, coronary angiography is performed with the 50% contrast mixture to delineate the left coronary anatomy identifying the LAD coronary artery and the second diagonal branch (D2). This will guide identification of the occlusion target which is in the LAD after the D2 branch.

15. A bolus dose of 100mg Lidocaine is administered intravenously 20-30 minutes prior to coronary occlusion via the peripheral cannula.

16. Coronary engagement is maintained with the guide catheter, whilst a 0.0014 in wire (normal length wire) is advanced down to the distal LAD. The wire is introduced into the haemostasis valve via the introducer needle provided in the set.

    The wire is advanced gently under fluoroscopic guidance to avoid coronary perforation. Avoid buckling the wire tip as it is advanced.

17. The indeflator is prepared with the 50:50 contrast and heparinised saline solution mix. For this, the chamber of the indeflator is filled with the mixture by aspiration, then the handle is turned clockwise to expulse the fluid, removing any leftover bubbles and leaving the mixture bubble-free.

18. Depending on the approximated diameter of the vessel (which is determined by comparing the vessel calibre to the guide catheter, which represents a width of approximately 2 mm), an appropriate size intracoronary balloon is selected.

19. The balloon's chamber is filled with contrast to ensure there is no air and is connected to the indeflator. A vacuum is created by adding negative pressure and holding it in place to

empty the balloon and ensuring that there is no air within. The vacuum must be maintained during the balloon's introduction.

⚠ Ensure that the device is undamaged.

Do not pre-inflate or test the balloon before insertion.

20. The protective sleeve is removed from the balloon. The balloon catheter has a central lumen, which allows it to be advanced over the 0.0014 in wire.
21. The 0.0014” wire is fixed in the distal LAD and the intracoronary balloon is advanced to the target site of occlusion over this wire whilst maintaining the distal position of this wire at all times.

⚠ As the balloon is advanced, it is critical to hold in place the thin wire, so it does not cause coronary trauma or perforation by inadvertent advancement.

22. The indeflator is used to inflate the intracoronary balloon at the target occlusion point within the LAD (i.e., immediately after the D2 vessel bifurcates). The contrast mixture is filled into the intracoronary balloon by the indeflator, resulting in the occlusion.

⚠ Inflation of the intracoronary balloon is performed slowly to ensure adequate but not excessive inflation which can cause coronary artery damage.

23. A coronary angiogram is performed to confirm that there is no visible flow of contrast distal to the occlusion point suggesting adequate balloon inflation and this inflation is maintained for a 90-minute duration.
24. A further bolus intravenous dose of 50mg Lidocaine is administered via the peripheral cannula at 20 minutes post occlusion.

⚠ Continuous monitoring of ECG, blood pressure, and oxygen saturations are necessary during this time.

25. Typically, animals begin to show ST changes almost immediately after the occlusion, with ventricular arrhythmias happening between 20-40 minutes later. Any ventricular arrhythmias need to be quickly treated by internal cardiac defibrillation using the 35J cardiac defibrillator device that has been implanted in the beginning of the procedure.

This is done manually, using the header of the ICD programmer, since the device is in emergency mode.

Prompt defibrillation is necessary upon recognition of ventricular arrhythmia. This may sometimes require multiple defibrillations. Therefore it is important to ensure that the implanted device has sufficient battery life.

26. Coronary perfusion is restored by deflation of the balloon at 90 minutes with a coronary angiogram confirming re-perfusion down the coronary artery.
27. Atenolol 1mg is administered intravenously via the peripheral cannula on reperfusion.
28. The guide, balloon and wire are removed from the heart.
29. The carotid sheath is removed and the carotid artery is tied off with a 2-0 silk suture achieving haemostasis.

The removal of the carotid sheath is performed simultanously as the carotid is tied off to avoid excessive bleeding.

30. The wound is closed in layers with a 2-0 monocryl absorbable suture.
31. The animal is then gradually awakened, extubated, and recovered and monitored until standing and consuming food and water.
32. The animal's welfare and post-operative progress is checked repeatedly following one hour after recovering from the procedure.
33. On the first day, any interaction with the animal is limited with observation from a short distance to prevent catecholamine mediated arrhythmias.
