# Supplementary material for: A refined, minimally invasive, reproducible ovine ischaemia–reperfusion–infarction model using implantable defibrillators: Methodology and validation
Source: Exp Physiol. 2024 Dec 19;110(2):215–29. doi: 10.1113/EP091760 (PMC11782204; doi:10.1113/EP091760)
Supplement: Supplementary file 2 — Supplementary videos 1–3 [file EPH-110-215-s002.pptx]

## Slide 1
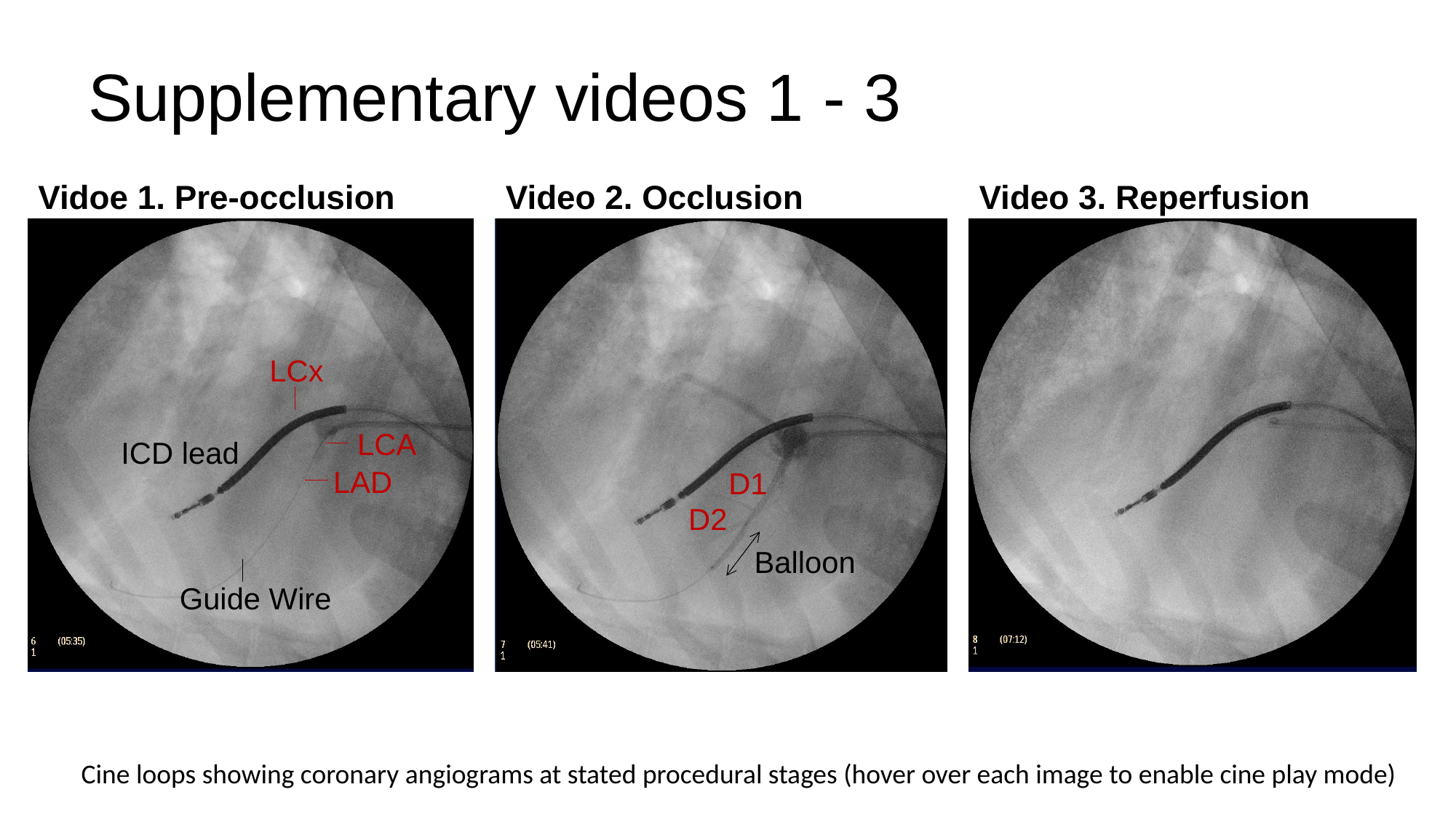

# Supplementary videos 1 - 3
Vidoe 1. Pre-occlusion
LCx
LCA
ICD lead
LAD
Guide Wire
Video 2. Occlusion
Video 3. Reperfusion
Weeks
D1
D2
Balloon
Cine loops showing coronary angiograms at stated procedural stages (hover over each image to enable cine play mode)
